# Total neoadjuvant immunochemotherapy for proficient mismatch repair or microsatellite stable locally advanced rectal cancer

**DOI:** 10.3389/fimmu.2025.1611386

**Published:** 2025-06-20

**Authors:** Xing Li, Ligong Tang, Fangyuan Cheng, Yongchao Xu

**Affiliations:** Department of General Surgery, The Affiliated Cancer Hospital of Zhengzhou University & Henan Cancer Hospital, Zhengzhou, China

**Keywords:** total adjuvant therapy, short-course radiotherapy, immunotherapy, locally advanced rectal cancer, watch-and-wait

## Abstract

**Objective:**

Our goal was to assess the efficacy of integrating PD-1 inhibitors with total neoadjuvant treatment (iTNT) in enhancing complete response (CR) rates and the propensity for watch-and-wait (WW) strategies in patients with proficient mismatch repair or microsatellite stable (pMMR/MSS) locally advanced rectal cancer (LARC).

**Methods:**

A retrospective analysis of data prospectively collected was performed. Enrolled patients were divided into Group SCRT-IC, which received short-course radiotherapy (SCRT) followed by six cycles of consolidation immunotherapy with capecitabine and oxaliplatin, or to Group IC-SCRT, which underwent two cycles of induction immunotherapy followed by SCRT and the remaining four cycles of chemotherapy. The primary endpoint was CR.

**Results:**

A total of 141 patients were included (72 in Group SCRT-IC and 69 in Group IC-SCRT). At a median follow-up of 29 months, the CR rates were 55.6% in Group SCRT-IC and 53.6% in Group IC-SCRT. The pCR rates were reported at 50% for both groups. Seventeen patients in each group were treated with WW and remained disease-free. The most prevalent grade 3 to 4 toxicities were thrombocytopenia and neutropenia. The cCR rate was a little higher in Group SCRT-IC (56.9% compared to 53.6%), and the incidence of grade 3 to 4 thrombocytopenia was lower in Group SCRT-IC (24.2% vs. 33.9%).

**Conclusion:**

iTNT regimen has significantly improved the CR rate for pMMR/MSS LARC compared to historical standards, with acceptable toxicity. The approach of prioritizing SCRT followed by immunotherapy is a promising strategy for definitive investigation in future studies.

## Introduction

Total neoadjuvant therapy (TNT) has been developed to improve compliance and efficacy of preoperative systemic treatment for locally advanced rectal cancer (LARC), especially in cases with early micrometastases. Numerous randomized clinical trials have demonstrated that TNT, encompassing neoadjuvant chemoradiotherapy, followed by total mesorectal excision (TME), and subsequent adjuvant chemotherapy, has emerged as the gold standard for the management of LARC, significantly contributing to tumor regression and a reduction in distant metastases. Nonetheless, the rate of pathological complete response (pCR) achieved with this approach is less than 30%, as documented in two expansive Phase III trials (1, 2). In light of the growing preference for non-surgical treatment options (NOM) to obviate the need for permanent colostomy in rectal cancer patients, there remains an imperative to investigate novel therapeutic modalities that may further augment the persistence of complete response (CR).

Immune checkpoint inhibitors (ICIs) that impede PD-1 have demonstrated remarkable efficacy, culminating in a significant uptick in clinical complete responses (cCR) reaching as high as 100% in patients with colorectal cancer (CRC) harboring mismatch repair defects (dMMR) or high microsatellite instability (MSI-H) (3). However, these agents are predominantly employed as monotherapy for the majority (approximately 85%) of CRC cases that possess proficient mismatch repair (pMMR) or microsatellite stability (MSS). When tested in the context of metastatic CRC, the integration of conventional therapeutic strategies with ICIs is being actively investigated to surmount the immune resistance encountered in pMMR/MSS CRC. The synergistic pairing of radiation therapy (RT) with ICIs has garnered considerable interest, buoyed by evidence supporting their interaction and mutual potentiating effects (4). Recently, numerous Phase I to II trial reports have emerged, indicating that the ICI-RT protocol has the potential to elicit tumor regression in pMMR/MSS LARC, with observations of a 30% pCR rate following protracted radiotherapy and chemotherapy. Additionally, 46.2% of patients who received short-course radiotherapy (SCRT) followed by nivolumab therapy, in conjunction with the combination of capecitabine and oxaliplatin (CAPOX) plus a capecitabine monoclonal antibody, achieved pCR (5). Nonetheless, the implications for organ preservation, particularly within the realm of NOM, have been infrequently examined.

In light of the benefits afforded by the TNT regimen and the potential for synergistic effects between radiation and ICIs, we postulate that immunotherapy-based TNT (iTNT) for the treatment of pMMR/MSS LARC, will manifest a higher CR rate than that historically achieved with conventional radiotherapy and chemotherapy-based TNT. Therefore, this study aimed to assess the combination of SCRT with CAPOX and ICIs as an immunochemotherapy approach for TNT in pMMR/MSS LARC. Given the uncertainty surrounding the optimal timing of ICI administration, either prior to or subsequent to radiation therapy, we have also evaluated the utilization of SCRT in conjunction with immunochemotherapy.

## Patients and methods

### Ethical approval

This study was approved by Henan Cancer Hospital Institutional Research Committee, and written informed consent for medical research was obtained from all patients before starting the treatment. All methods were performed in accordance with the relevant guidelines and regulations.

### Study design

**Figure 1:**
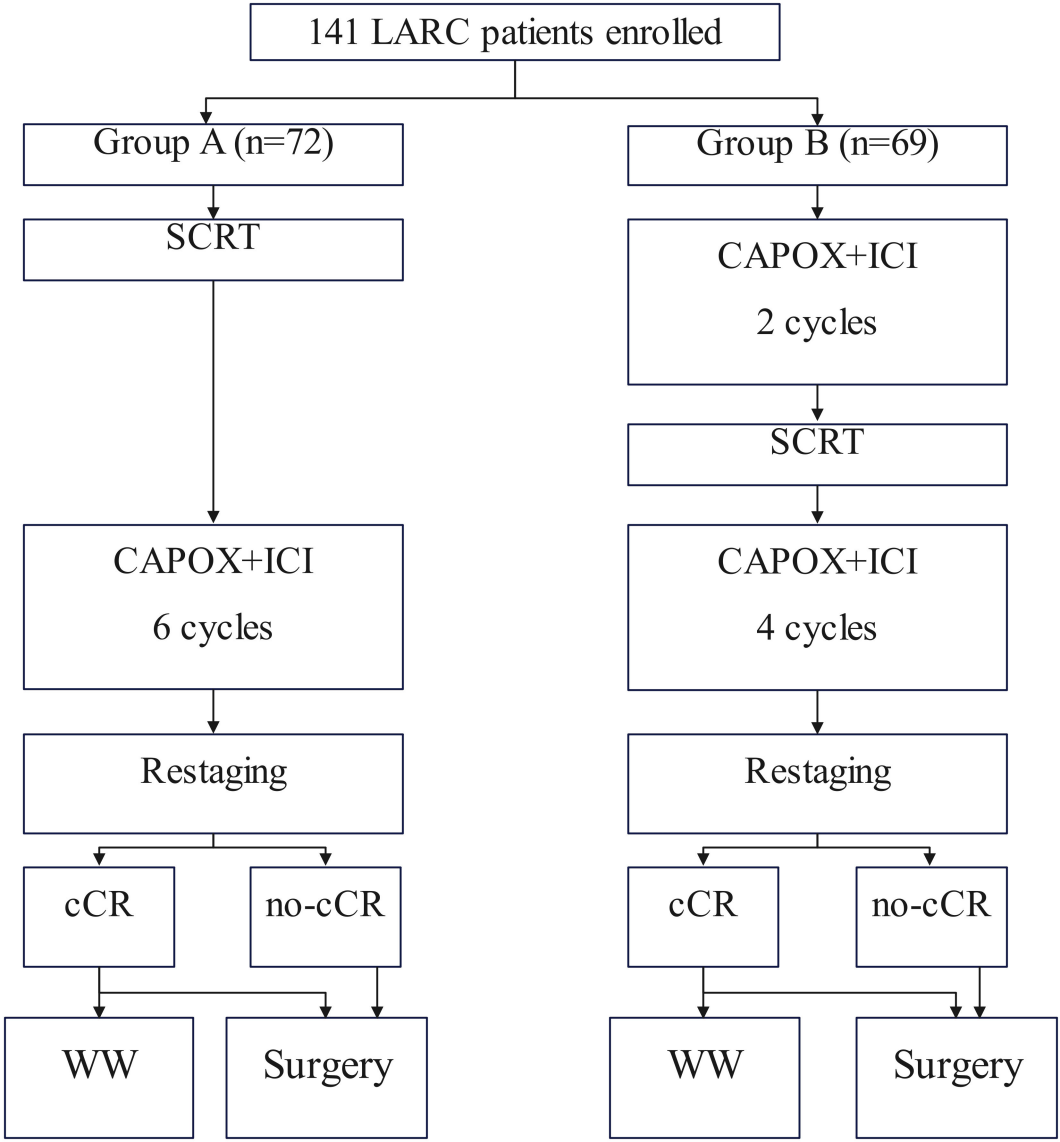
Treatment details of the enrolled patients.

To address this purpose, a retrospective analysis of data prospectively collected was performed in a tertiary hospital between January 2021 and December 2024. Medical records of patients with primary rectal adenocarcinoma located ≤12cm from the anal verge was reviewed. Enrolled patients must meet the following criteria: the disease was staged as cT3/4N0 or cT_any_N1/2 based on the 8^th^ AJCC system; iTNT was conducted (**Figure 1**). Patients exhibiting a prior cancer history were excluded. Data regarding demography, pathology, treatment, and follow-up was analyzed.

### Treatment

These patients were divided into two groups based on different immunotherapy program. In one cohort (Group SCRT-IC), patients received SCRT followed by six cycles of consolidation immunochemotherapy and the other population (Group IC-SCRT) underwent two cycles of induction immunochemotherapy followed by SCRT and the rest four doses (6). SCRT is administered at a dose of 25 Gy fractionated over five sessions, utilizing intensity-modulated radiotherapy to target both the primary tumors and the regional pelvic lymph nodes. Concurrently, we have enhanced the immunotherapy component by combining CAPOX with PD-1 inhibitors consisting of Pembrolizumab, Toripalimab, and Tislelizumab, which involves administering 240 mg of ICIs once daily on the first day, alongside oxaliplatin at a dose of 130 mg/m²as a single daily dose, and capecitabine at a dose of 1000 mg/m²administered twice daily from day 1 to day 14. This treatment regimen is repeated every 21 days, comprising a single cycle.

Tumor re-staging is conducted within four weeks following the completion of TNT, with radiological assessment performed via digital rectal examination (DRE), endoscopic evaluation, MRI of the rectum, and contrast-enhanced CT scans of the chest, abdomen, and pelvis. Individuals who have achieved a cCR after treatment are placed on a standardized watch-and-wait (WW) protocol. This WW protocol encompasses DRE, enhanced imaging, and endoscopic surveillance every 8-12 weeks during the initial two years, followed by monitoring every 3-6 months for the subsequent three years. It is advisable for patients to undergo an examination for signs of tumor regrowth post-TME. For patients who have not achieved cCR, a subsequent investigation in accordance with current guidelines is recommended following the restart of TME (7). Adjuvant chemotherapy after curative surgery was not recommended.

### Variable definition

The primary endpoint is the CR rate, which is computed by dividing the number of patients who achieved a pCR by the sum of patients who underwent surgery after WW and those who achieved a cCR, all based on the total number of assessable patients. Pathological tumor regression is classified utilizing the refined Ryan schema, with the tumor regression grade (TRG) assessed by estimating the percentage of viable tumor cells within a grossly visible tumor bed (8). A pCR is defined as the absence of any tumor cells in the primary tumor and lymph nodes following radical surgery (ypT0N0), or the lack of tumor cells in the lesion after local excision surgery (ypT0). A cCR is defined as the absence of residual disease as determined by digital rectal examination, MRI, and endoscopic examination. Secondary endpoints include toxicity, adherence to treatment, surgical complications, and long-term outcomes, as previously outlined. Adverse events and surgical complications are classified in accordance with the Common Terminology Criteria for Adverse Events (CTCAE), Version 5.0 (9).

### Statistical analysis

The comparison of the CR rate is conducted utilizing the Z-test, which employs a normal approximation method. An exploratory comparative analysis of two sets of variables is performed using the chi-square test. To elucidate the significant factors associated with CR status, both univariate and multivariate logistic regression models are employed, incorporating treatment group, age, gender, distance from the anal margin, clinical stage, clinical classification T, clinical classification N, extramural vessel infiltration, and mesorectal fascia status. All analytical procedures were carried out using R 4.3.1, a statistical software platform, based in Vienna, Austria, for statistical computation.

## Results

### Baseline information

A total of 141 patients were enrolled, with an average age of 50 ± 14 years. The cohort comprised 101 males and 40 females. Of these, 107 tumors were situated within 5 centimeters of the anal verge. The clinical staging was as follows: 16 patients were classified as stage II, while 125 patients were classified as stage III. Clinical evidence of extramural vascular invasion and mesorectal fascia invasion was observed in 36 and 44 patients, respectively. Seventy-two patients were assigned to Group SCRT-IC, and they exhibited a comparable demographic and radiological profile to those in Group IC-SCRT, with no significant differences between the groups (all p-values > 0.05, **Table 1**).

**Table 1:** Baseline data of these patients.

Variable	Total (n=141)	Group SCRT-IC (n=72)	Group IC-SCRT (n=69)	P*
Age				
≤50	88	42	46	
>50	53	30	23	0.307
Sex				
Male	101	48	53	
Female	40	24	16	0.182
Distance from anal verge (cm)				
≤5	107	54	53	
>5	34	18	16	0.802
Clinical stage				
II	16	6	10	
III	125	66	59	0.249
cEMVI^$^				
Negative	105	55	50	
Positive	36	17	19	0.593
cMRF^				
Negative	97	53	44	
Positive	44	19	25	0.207

* Comparison between group A and B;

$ EMVI, extramural vascular invasion;

^ MRF: mesorectal fascia.

### Treatment compliance and toxicity

All patients in both cohorts (100%) received the prescribed radiotherapy dosage (25Gy/5 fractions). Sixty-three individuals (87.5%) in Group SCRT-IC and 63 (91.3%) in Group IC-SCRT completed at least five cycles of CAPOX in conjunction with ICI treatment, with 53 (73.6%) and 59 (85.5%) patients, respectively, completing all six cycles, as detailed in **Table 2**. No significant discrepancies were noted in terms of treatment adherence between the two groups. Thirty-two patients (44.4%) in Group SCRT-IC and 29 (42.0%) in Group IC-SCRT experienced grade 3 and 4 adverse reactions, as documented in **Table 3**. The prevalent grade 3 to 4 adverse reactions included thrombocytopenia (23.6% in Group SCRT-IC and 33.3% in Group IC-SCRT) and neutropenia (11.1% in Group SCRT-IC and 5.8% in Group IC-SCRT). Two patients in Group IC-SCRT succumbed to causes unrelated to the treatment prior to the scheduled surgery: one in a vehicular accident and the other due to cerebral infarction, a complication of hypertension and diabetes.

**Table 2:** Treatment compliance with SCRT* and immunotherapy.

Feature	Group SCRT-IC (n=72)	Group IC-SCRT (n=69)
Compliance with SCRT	72 (100%)	69 (100%)
Compliance with immunotherapy		
Compliance with CAPOX + ICI^%^		
Completed 6 cycles	53 (73.6%)	59 (85.5%)
Completed 5 cycles	10 (13.9%)	4 (5.8%)
Completed ≤4 cycles	9 (12.5%)	6 (8.7%)
Compliance with oxaliplatin		
Completed 6 cycles	58 (80.6%)	61 (88.4%)
Completed 5 cycles	7 (9.7%)	5 (7.2%)
Completed ≤4 cycles	7 (9.7%)	3 (4.3%)
Compliance with ICI		
Completed 6 cycles	56 (77.8%)	62 (89.9%)
Completed 5 cycles	9 (12.5%)	1 (1.4%)
Completed ≤4 cycles	7 (9.7%)	6 (8.7%)
Compliance with capecitabine		
Completed 6 cycles	64 (88.9%)	67 (97.1%)
Completed 5 cycles	6 (8.3%)	2 (2.9%)
Completed ≤4 cycles	2 (2.8%)	0
Interval time (weeks)		
From the start of iTNT to restaging^#^	23.4 ± 3.0	23.9 ± 2.7
From the end of SCRT to restaging	22.5 ± 3.0	17.2 ± 2.5
From the end of iTNT to restaging	2.6 ± 0.4	2.7 ± 0.5

^*^ SCRT: short-course radiotherapy;

^%^ CAPOX: capecitabine and oxaliplatin; ICI: immune checkpoint inhibitor;

^#^ iTNT: immunotherapy-based total neoadjuvant therapy.

**Table 3:** Acute adverse events of iTNT during neoadjuvant treatment.

Events	Group SCRT-IC (n=72)		Group IC-SCRT (n=69)	
	G1/2	G3/4	G1/2	G3/4
Thrombocytopenia	31 (43.1%)	17 (23.6%)	24 (34.8%)	23 (33.3%)
Leukopenia	37 (51.4%)	5 (6.9%)	36 (52.2%)	3 (4.3%)
Neutropenia	30 (41.7%)	8 (11.1%)	31 (44.9%)	4 (5.8%)
Anemia	36 (50.0%)	2 (2.8%)	32 (46.4%)	4 (5.8%)
ALT elevation	25 (34.7%)	3 (4.2%)	31 (43.1%)	2 (2.9%)
AST elevation	34 (47.2%)	3 (4.2%)	35 (50.7%)	2 (2.9%)
TBIL elevation	17 (23.6%)	0	10 (14.5%)	0
Cr elevation	11 (15.3%)	0	6 (8.7%)	0
TSH elevation	9 (12.5%)	0	10 (14.5%)	0
cTnT elevation	6 (8.3%)	0	9 (13.0%)	0
CKMB elevation	4 (5.6%)	0	6 (8.7%)	0
proBNP elevation	11 (15.3%)	0	12 (17.4%)	0
Fatigue	60 (83.3%)	3 (4.2%)	57 (82.6%)	2 (2.9%)
Poor appetite	43 (59.7%)	7 (9.7%)	58 (84.1%)	4 (5.8%)
Nausea	50 (69.4%)	3 (4.2%)	62 (89.9%)	1 (1.4%)
Vomiting	28 (38.9%)	3 (4.2%)	29 (42.0%)	2 (2.9%)
Constipation	23 (31.9%)	0	27 (39.1%)	0
Tenesmus	44 (61.1%)	0	59 (85.5%)	0
Hand-foot syndrome	22 (30.6%)	0	25 (36.2%)	0
Peripheral neurotoxocity	55 (76.4%)	4 (5.6%)	60 (87.0%)	2 (2.9%)
Hypothyroidism	9 (12.5%)	0	11 (15.9%)	0
Pneumonia	4 (5.6%)	0	3 (4.3%)	0
Colitis	2 (2.8%)	1 (1.4%)	2 (2.9%)	1 (1.4%)
Vitiligo	2 (2.8%)	0	4 (5.8%)	0

At the restart, 41 patients (56.9%) in Group SCRT-IC and 37 (53.6%) in Group IC-SCRT achieved a clinical complete response (cCR). Seventeen patients from each group elected to proceed with WW surgery, while the remainder opted for surgery. Among the patients who did not attain cCR, 9 cases in Group SCRT-IC and 12 patients in Group IC-SCRT refused surgery. Ultimately, 46 patients in Group SCRT-IC and 40 in Group IC-SCRT underwent surgical intervention. The duration of treatment, as well as the interval between treatments and from start to finish, was comparable between the two groups. Notably, Group SCRT-IC exhibited a protracted interval from chemoradiotherapy (SCRT) to surgery resumption, in accordance with the study’s design, as outlined in **Table 2**.

### Efficacy

The median follow-up period was 29 months, with a range of 11 to 47 months. Of the patients who underwent WW, 34 (17 in each group) maintained a cCR during the recent follow-up, with 19 patients sustaining cCR for over 12 months. One patient from Group SCRT-IC and one from Group IC-SCRT experienced tumor recurrence at the 10th and 7th month of WW, respectively, and subsequently underwent rescue total mesorectal excision (TME) with sphincter preservation surgery. Among the 86 patients who underwent surgery, 50% (23 of 46 cases in Group SCRT-IC and 20 of 40 cases in Group IC-SCRT) achieved a pCR. Consequently, the CR rate was 55.6% (17 cCR plus 23 pCR divided by 72 cases in Group SCRT-IC, and 53.6% (17 cCR plus 20 pCR divided by 69 cases in Group IC-SCRT). There was no significant difference in CR rate between the two observation treatment groups based on univariate logistic regression analysis. Multiple logistic regression analysis indicated that early onset age (OR, 2.45 [95% CI, 1.32 to 6.89]; P=0.008) was an independent risk factor for an incomplete interval TME response after adjusting for model variables (**Supplementary Table 1**).

For patients who underwent surgery, complete resection (R0) was achieved in 46 patients in Group SCRT-IC (100%) and 39 in Group IC-SCRT (97.5%), with one case in Group IC-SCRT (2.5%) exhibiting a circumferential resection edge of ≤1mm. Sphincter preservation surgery was performed on 33 patients (71.7%) in Group SCRT-IC and 33 patients (82.5%) in Group IC-SCRT (**Table 4**). **Figure 2** illustrates the percentage of histopathological tumor regression for each group, with major pathological regression (≤10% residual viable tumor) observed in 67.4% of patients in Group SCRT-IC and 70.0% in Group IC-SCRT. Forty-two patients (91.3%) in Group SCRT-IC and 32 (80.0%) in Group IC-SCRT reported negative lymph nodes. Postoperative grade 3-4 complications included one vaginal fistula and one anal fistula in Group SCRT-IC, with no grade 3 to 4 wound infections, arterial stenosis, or intestinal obstruction noted. No mortalities occurred within 60 days post-surgery. There was no significant difference between the groups in terms of grade 3 to 4 surgical complications. At the conclusion of the follow-up, the anal sphincter was successfully preserved in 59 patients (81.9%) in Group SCRT-IC and 62 (89.9%) in Group IC-SCRT.

**Table 4:** Efficacy of iTNT, surgical, and pathologic results in these patients.

Feature	Group SCRT-IC (n=72)	Group IC-SCRT (n=69)	p
Overall efficacy			
CR	41 (56.9%)	37 (53.6%)	0.692
WW with continuous cCR	17	17	
Surgical efficacy			
Number of patients received surgery	46	40	
Surgical procedure			
Anterior resection	32	30	
Abdominoperineal resection	10	7	
Local excision	2	2	
Hartmann procedure	2	1	
R0 resection			
Yes	46 (100%)	39 (97.5%)	
No	0	1 (2.5%)	0.465
Sphincter-sparing surgery			
Yes	33 (71.7%)	33 (82.5%)	
No	13 (28.3%)	7 (17.5%)	0.239
Grade 3/4 surgical complication			
Anastomotic fistula	1 (2.2%)	1 (2.5%)	1.000
Vaginal fistula	1 (2.2%)	1 (2.5%)	1.000
Pathologic efficacy			
pCR			
Yes	23 (50.0%)	20 (50.0%)	
No	23 (50.0%)	20 (50.0%)	1.000
TRG score*			
0	23 (50.0%)	20 (50.0%)	
1	6 (13.0%)	7 (17.5%)	
2	14 (30.4%)	6 (15.0%)	
3	3 (6.5%)	7 (17.5%)	0.201
ypT			
0	23 (50.0%)	20 (50.0%)	
1	2 (4.3%)	2 (5.0%)	
2	15 (32.6%)	8 (20.0%)	
3	5 (10.9%)	8 (20.0%)	
4	1 (2.2%)	2 (5.0%)	0.585
ypN			
0	42 (91.3%)	32 (80.0%)	
1	4 (8.7%)	6 (15.0%)	
2	0	2 (5.0%)	0.178
ypEMVI^			
Negative	44 (95.7%)	39 (97.5%)	
Positive	2 (4.3%)	1 (2.5%)	1.000
ypPNI^&^			
Negative	42 (91.3%)	34 (85.0%)	
Positive	4 (8.7%)	6 (15.0%)	0.504
CRM^#^			
Negative	46 (100%)	39 (97.5%)	
Positive	0	1 (2.5%)	0.465

* TRG: tumor regression grade;

^ EMVI: extramural vascular invasion;

& PNI: perineural invasion;

# CRM: circumferential resection margin.

**Figure 2:**
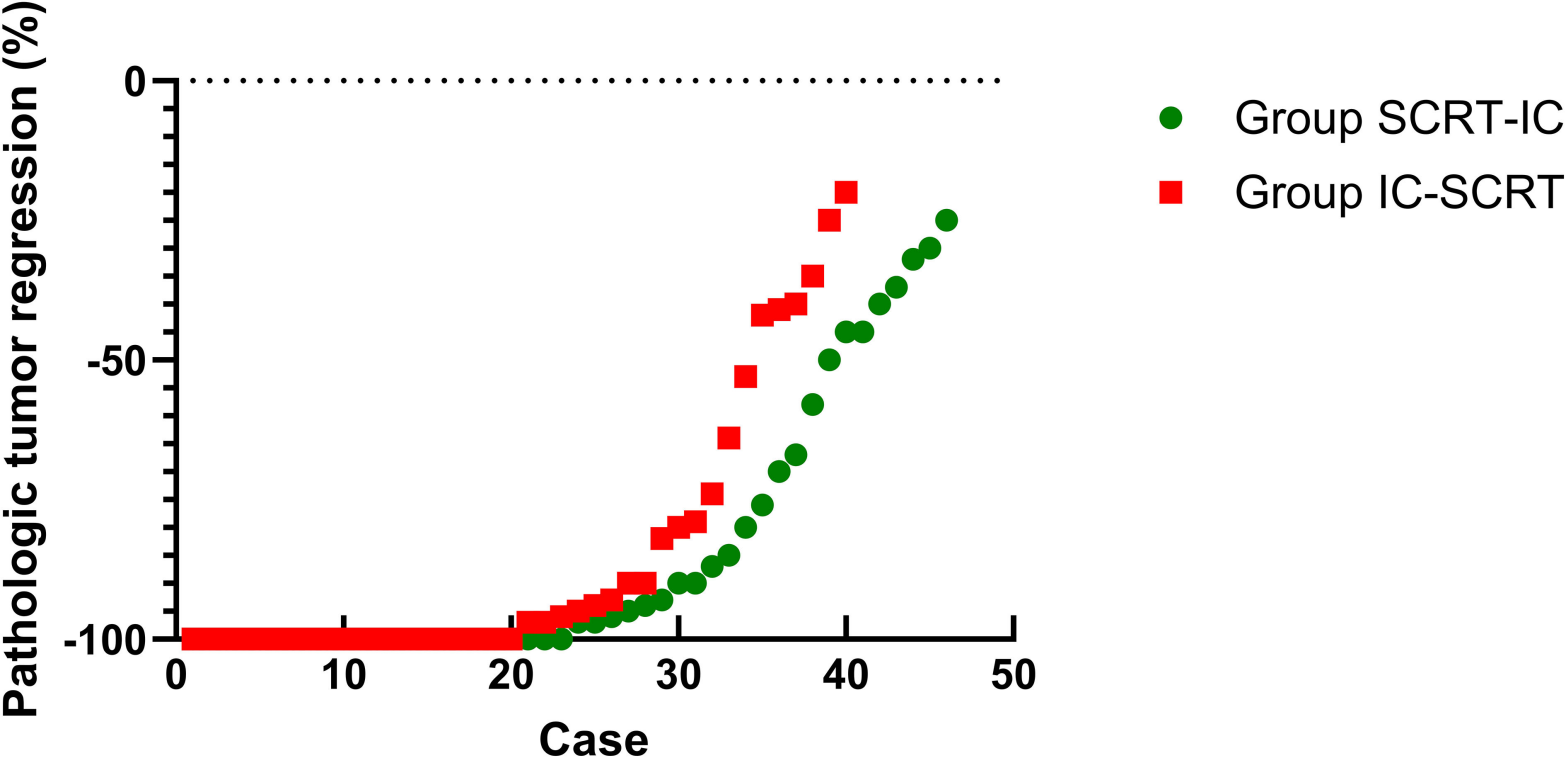
Pathologic tumor regression in Group SCRT-IC and Group IC-SCRT.

## Discussion

To our knowledge, we are pioneering the first investigation into the efficacy and safety of a novel therapeutic approach, integrating immunotherapy-modulated total neoadjuvant therapy with selective WW strategies in patients with pMMR/MSS LARC. This study has validated the predetermined statistical hypothesis, demonstrating that the CR rate of iTNT is superior when compared to historical controls, despite variations in the scheduling of SCRT and immunotherapy. Considering that 80% of the patients enrolled in this study present with tumors in the lower rectum, our findings suggest that the iTNT methodology possesses significant potential to emerge as a promising treatment option for organ preservation within the pMMR/MSS LARC patient population.

The CR rate observed in current study significantly surpasses that of the pCR or cCR rate in the POLISH II experimental group (10), as well as the rates reported in the RAPIDO (2) and STELLAR trials (11), which utilized a similar TNT approach based on SCRT. The most eagerly anticipated explanation for this improvement is the potential enhancement of tumor regression with the addition of immunotherapy. In concordance with our findings, a phase III trial (UNION) presented by Lin et al. at the 2023 European Medical Oncology Society Conference reported that following SCRT (12), two cycles of camrizumab and CAPOX resulted in a pCR rate that surpassed that of local chemoradiotherapy, with subsequent cycles of CAPOX alone (39.8% vs. 15.3%). Notably, the NRG-GI00226 trial demonstrated that the addition of pembrolizumab to radiotherapy and chemotherapy in a TNT setting significantly improved the 3-year overall survival rate, although no significant difference was found in the use of new adjuvants for rectal scoring (13). These outcomes are in alignment with our analysis, collectively underscoring the additional benefits of ICIs in the pMMR neoadjuvant therapy context. Another plausible explanation could be the difference in endpoint evaluation. We reported the combined pCR and cCR rate, whereas RAPIDO and POLISH II primarily reported pCR rates (2, 10), which may result in an underestimation of the overall CR in cases where surgery was performed before achieving cCR. For instance, in the OPRA trial (14), the intentional treatment of cCR/near cCR with watch-and-wait led to the maintenance of cCR in as high as 43% to 55% of patients. Additional factors include patient characteristics, such as the lower proportion of high-risk features of T4 tumors and N2 disease, which may also contribute to the higher CR rate observed in our cohort. As previously stated, confirmatory evidence of the our results requires a large-scale phase III trial to investigate the iTNT arm against a non-immunotherapy TNT arm, employing a robust study design.

Current study has enriched the existing body of knowledge by establishing the preliminary viability of the WW approach in pMMR/MSS patients who undergo iTNT based on SCRT. To date, total neoadjuvant therapy founded on long-course radiotherapy (LCRT) appears to be the preferred choice when the objective is to achieve maximal tumor regression and no evidence of residual disease (NOM). Two prospective studies that combined ICI with LCRT have reported a cCR rate of 31% to 43.5% in pMMR/MSS LARC patients, yet these studies involved patients with less aggressive disease (T1-3aN0-1) and did not provide information on the follow-up with a watch-and-wait strategy (13, 15). The role of SCRT as a precursor to a WW approach remains a topic of debate, with lingering concerns about the relatively low biologically effective dose of SCRT, as per the linear quadratic model, which has been associated with an increased risk of local failure as seen in the RAPIDO trial (2). Despite these considerations, our research findings underscore the robust tumor regression effect achieved through the combination of SCRT and ICIs. This efficacy may be at least partially attributed to the superior characteristics of SCRT compared to LCRT, including the synergistic effects of radiotherapy and ICIs, such as the induction of mild treatment-associated lymphopenia, enhanced antigen release, and an increase in tumor-infiltrating lymphocytes (16). It has been noted that when combined with pembrolizumab, a radiation dose of 50 Gy in 4 fractions can elicit a superior response rate compared to 45 Gy in 15 fractions (48% vs. 25%). Additionally, our tumor recurrence rate is significantly lower than that observed in the OPRA trial (36%), which may be due to the relatively short follow-up period and the stringent inclusion criteria that only permit patients who achieve cCR with the WW approach. This also accounts for OPRA patients who are near cCR. An intriguing explanation is that, as is characteristic in dMMR/MSI-H CRC, the response to ICIs exhibits persistent efficacy. Long-term follow-up and correlative analyses are necessary to substantiate this hypothesis.

In current study, the overall CR rates among the two patient cohorts, those commencing with SCRT or immunotherapy (17, 18), were found to be comparable, which can be ascribed to the similar overall treatment intensity and duration. Notably, Group SCRT-IC, which prioritized SCRT, exhibited a higher cCR rate (56.9% versus 53.6%), potentially due to a prolonged interval to completion. Moreover, the incidence of grade 3 to 4 thrombocytopenia was lower in the SCRT group (23.6% versus 33.3%) during neoadjuvant therapy. Although a chi-square test revealed no statistically significant differences between the two groups, this may be because the trial did not detect substantial disparities. Nevertheless, these findings imply that the initial use of SCRT may be correlated with an augmented opportunity for WW strategies and a diminished risk of serious adverse events. Our results, in conjunction with the findings from prior studies that examined the sequence of chemoradiotherapy and chemotherapy (19), suggest that initiating with radiotherapy may either result in a higher CR rate or achieve organ preservation with a toxicity level of grade 3 or less. Consequently, the selection of Group SCRT-IC, commencing with SCRT followed by consolidation immunotherapy, warrants further investigation in phase III trials.

It is intriguing to note that the current study revealed that some patients who did not meet the criteria for cCR were confirmed to have a pCR upon pathological examination. This occurrence, termed pseudoresidual disease, may be more prevalent than previously recognized. A recent study reports (20) that in locally treated dMMR/MSI-H CRC patients who received neoadjuvant ICIs, 72% and 42% of patients exhibited residual disease on imaging and endoscopy, respectively, with no evidence of progression during pCR or WW protocols. This compares to only 8.3% to 16.6% of patients experiencing pseudorecurrence following radiotherapy and chemotherapy. The insufficiency of tumor regression stimulation may lead to misleading treatment decisions and preclude patients from opportunities for organ preservation. Further research is imperative to enhance diagnostic precision and to establish a predictive model for identifying potential tumor responses and candidates for no evidence of residual disease.

Our findings indicate that about 80.0% of patients tolerate iTNT well, completing all prescribed plans, which compares favorably to the traditional TNT method. The overall rate of grade ≥ 3 toxicity is 43.2%, which is similar to that observed in the RAPIDO (47.6%) (2) and PRODIGE-23 experimental groups (46%) (1). However, the incidence of grade 3-4 thrombocytopenia in current study patients is relatively high at 28.4%, a finding also noted in trials investigating the combination of ICIs with oxaliplatin-based chemotherapy (12, 13). Due to the heightened risk of severe bleeding and the frequent treatment delays caused by severe thrombocytopenia, close monitoring during the treatment period is essential to ensure timely intervention. Moreover, extra caution should be exercised when applying this approach to patients with pre-existing thrombocytopenia as a result of underlying conditions. Additional research is required to elucidate this issue and to identify biomarkers predictive of toxicity.

Limitation in current study must be acknowledged, first, there was lack of randomization, it increased our selective bias; second, our sample size was relatively small, it might decrease our statistic power; third, this was a single-center design limited by relatively short follow-up, further clarification on long-term toxicities, biomarker-driven stratification, and external validation was needed.

In summation, iTNT paradigm has the potential to facilitate complete response in over half of pMMR/MSS patients with locally advanced rectal cancer, while maintaining an acceptable level of toxicity. Our research affords a promising solution that allows pMMR patients to undergo no evidence of residual disease protocols for MSS LARC, warranting further validation. The approach of consolidating SCRT with immunotherapy demonstrates enhanced efficacy and a favorable safety profile, having been selected for investigation in phase III trials.

## Data Availability

The original contributions presented in the study are included in the article/**Supplementary Material**. Further inquiries can be directed to the corresponding author.
