# Supplementary material for: Total neoadjuvant immunochemotherapy for proficient mismatch repair or microsatellite stable locally advanced rectal cancer
Source: Front Immunol. 2025 Jun 20;16:1611386. doi: 10.3389/fimmu.2025.1611386 (PMC12226533; doi:10.3389/fimmu.2025.1611386)
Supplement: Supplementary file 1 [file Table1.doc]

| Variable | Univariate | Multivariable | |
| --- | --- | --- | --- |
|  | p | OR [95%CI] | p |
| Age (>50 vs ≤50) | 0.013 | 2.45 [1.32-6.89] | 0.008 |
| Sex (Male vs female) | 0.328 |  |  |
| Distance from anal verge (>5cm vs ≤5cm) | 0.512 |  |  |
| Clinical stage (Ⅲ vs Ⅱ) | 0.443 |  |  |
| cEMVI^$^ (Positive vs negative) | 0.014 | 1.34 [0.73-3.76] | 0.276 |
| cMRF^ (Positive vs negative) | 0.023 | 1.13 [0.52-4.00] | 0.307 |
| Treatment (Group IC-SCRT vs Group SCRT-IC) | 0.634 | 0.97 [0.38-2.15] | 0.534 |

Supplementary Table 1. Univariate and multivariable analysis of predictors for CR status.

* Comparison between group A and B;

$ EMVI, extramural vascular invasion;

^ MRF: mesorectal fascia.
